# Fracture Don’ts

**Published:** 1917-10

**Authors:** Frank T. Fort

**Affiliations:** Louisville, Ky.


### FRACTURE DON'TS.

**Frank T. Fort, M.D., Louisville, Ky.**

A rather careful scrutinity of available surgical literature fails to reveal a single recent contribution to the subject of "Francture Don'ts." The importance of this subject was brough forcibly to my mind several years ago, while assistant in the clinics of the late John B. Murphy, where we have the opportunity of seeing many unfavorable results of improperly treated and neglected fractures, such as ischemic myositis deformities and resulting nocrosis from impacted fractures, almost functionless arms and legs from fractures near the neck of the humerus, from Colles' fracture, Pott's fracture, angulation of the femur and tibia from faulty adjustment, etc., all of which could probably have been prevented by the application of proper treatment at the time of injury, plus the co-operation of the patient who is often at fault.

The two most important underlying principles in the treatment of fractures may be stated as: (1) Correction of the deformity by proper reduction of the fragments; (2) Retention of the broken bones in correct anatomic position by suitable dressing or apparatus until union has occurred. There are three essential factors in the successful treatment of fractures: (a) mechanical ingenuity, (b) anatomic knowledge, and (c) conservatism fortified by experience.

I believe we should treat all injuries, where fracture is suspected, as a fracture, until the x-ray examination or the development of symptoms warrant the exclusion of fracture. It is my opinion, however, that the more conservative the surgeon is, the better will be his results; the operative work should be left for those who from experience and special adaptability are better qualified to secure favorable ultimate results,—I mean by this such procedure as bone plating, bone inlay, the use of nails, screws, etc.

Don't forget to dress a compound fracture in such manner that drainage can be instituted at any time, should the symptoms indicate or demand it.

Don't forget to arrange all the fractured bones in their of Paris dressings in the treatment of fractures must be acquired by every surgeon; it is not as easy and simple as one might think, it can only be learned by experience, the main objects being to secure fixation and rest without undue pressure.

Don't forget that a general anesthetic will cause muscular relaxation and facilitate the reduction of fracture in many instances where without its use reduction seemed impossible.

Don't attempt to apply any one kind of splint in a given fracture. Devise the kind of splint suitable for the individual case.

Don't forget to arrange all the fracture bones in their natural plane, with as much "muscle pull" removed as possible near the fracture site.

Don't forget that crepitus may be absent in: (a) riding of the fragments, (b) impaction of the fragments, (c) entire separation of the fragments, and (d) when muscular or blood clot is interposed between the fragments.

Don't forget that there is a pseudo-crepitus (very like true crepitus) in teno-synovitis, joint effusion, osteo-arthritis, and caries of joint surfaces.

Don't forget that in epiphyseal fracture the prognosis must be guarded, because such injuries in the young are sometimes followed by suspended growth or premature ossification followed by deformity.

Don't forget that in separation of the epiphysis (upper extremity of humerus and lower extremity of femur), the line of fracture is so broad that there may be no shortening, but the fragments may project.

Don't forget to at once examine the pulse at the wrist and ankle in fractures of the humerus and femur, to ascertain if an artery has been injured.

Don't allow a splint to press upon the skin sufficiently to produce ulceration or edema,—or what is worse,—gangrene.

Don't place a pad in the axilla or bandage the arm too tightly to the chest in fracture of the acromion, because the head of the humerus is thrown outward and may thus separate the fragments,— the head of the humerus is a natural splint in such fractures.

Don't forget to examine the shoulder joint in all fractures of the upper portion of the humerus, to ascertain whether the head is dislocated.

Don't begin passive motion too soon in fracture involving a joint, for it is likely to increase the formation of callus and thereby limit future usefulness of the joint; it is better to nail the fragments together and place the joint at complete rest.

Don't splint the palm of the hand in Colles' fracture; leave the fingers free so they may be "worked" after the second or third day, otherwise the tendons may become adherent where they cross the site of fracture and considerable time may be required to restores suppleness.

Don't make the diagnosis of "only a contused hip" in elderly people, without a careful and gentle examinaion (including the x-ray) to exclude impacted fracture.

Don't forget that rarely disintegration and absorption of the head and neck of the femur may occur in elderly persons as the result of chronic osteo-arthritis, which may simulate fracture in the shortening, eversion and osteophytic crepitus which are oftentimes present.

Don't use violence in attempting to elicit crepitus in hip fracture, as much damage may be inflicted by separating the impaction.

Don't keep elderly patients in bed trying to secure union in hip fractures; they are almost sure to develop pulmonary edema, pneumonia, sloughing from pressure of splints, or from bed sores, and nearly all of them die.

Don't forget to bandage the entire limb in fracture of the femur. I prefer the plaster of Paris cast reinforced with strips of tin, or the moulded plaster dressing. The splint should encase the foot, leg, thigh and pelvis, and a Buck's extension should be used.

Don't forget the danger to the popliteal artery from traction and extension in a transverse fracture of the femur above the condyle; an almast right-angle fracture box or splint is the best fixation dressing.

Don't place recent fractures in plaster of Paris without due regard to swelling; either place a wire saw underneath the circular plaster of Paris bandage, or cut it through from end-to-end when first applied, so it can be removed or readjusted when the swelling subsides; or what I like better in a great many fractures, apply moulded splints which can be made any shape desired while wet and can be removed much more easily than the circular type.

Don't forget that should severe pain develop within a few hours after a splint has been applied, the splint is either too tight from swelling, or the fracture has not been properly reduced,—excepting in cases where a severe sprain complicates the fracture; if pain is caused by swelling and the splint be not removed, within a few hours degenerative changes are likely to occur in the muscle cells which may induce ischemic myositis or (in the forearm) Volkmann's contracture. I have seen four or five such cases.

Don't forget to suspect degenerative changes due to syphilis, central sarcoma or other pathologic process producing friabiility of the bone, when fracture is produced by stepping upon a pebble or other slight violence.

Don't forget that in such instances there is usually a certain degree of anesthesia of the soft structures of the involved limb, and it may be difficult to prevent the patient walking too soon if a leg is fractured; and if the fracture is near a joint the condition may resemble a Charcot joint.

Don't fail to have an x-ray plate made in all fractures where perfect reduction seems doubtful, and this means nearly all of them; it is a valuable means of confirmation, and may be of great benefit to the surgeon should the result be unfavorable.

Don't attempt to plate a recent compound fracture, otherwise amputation will most likely be the inevitable result.

Don't use a plate in any recent fratcure (week or ten days) until all means at hand have been exhausted in attempted reduction.

Don't forget that in plating or inlay work the strictest asceptic technic must be used, not even the gloved hand should come in contact with the wound or the wound-touching portion of the instruments.

Don't place too much reliance upon the x-ray plate in suspected fractures near the shoulder joint, and in fractures at the angle of the lower jaw, as good pictures of these localities are difficult to obtain.

Don't forget the painful passive motion is always harmful where fracture involves a joint.

Don't forget in placing any kind of a splint around or to the outer side of the knee, to pad well over the head of the fibula, as pressure is likely to injure the peroneal nerve, produsing paralysis of the muscles supplied by it.

Don't forget to pad liberally under the heel of any splint that envelopes the foot, as pressure necrosis sometimes occurs. I have seen one such case in consultation following fracture of the patella, and healing was delayed for several months.

Don't forget to pad well over all bony prominences where splints have to be adjusted over them.

Don't forget that a burning sensation in the heel or other parts underneath a splint signifies that too much pressure is exerted; the splint should be immediately removed.

Don't forget that unless a fracture is complicated by a sprain, or severe contusion, when properly reduced and splinted pain should cease.

Don't forget the danger of injury to the anterior crural nerve in fracture of the true pelvis, since the fracture is most commonly through the ascending ramus of the ospubis at or near the point where the nerve crosses the bone; it is also likely to be injured in fractures and dislocations of the femur.

Don't forget that in fracture of the humerus there is danger of the musculo-spiral nerve being torn, and there is also danger of callus from the fracture interfering with the nerve.

Don't forget that in shoulder injuries complicated by dislocation, it is best not to attempt reduction of the dislocation until it has been ascertained no ribs have been fractured, for great damage might be inflicted upon the lung tissue should there be a broken rib.

Don't give a too favorable prognosis in any cranial fracture, for one never knows the extent of the crack in the skull, and infection may develop many days after the injury.

Don't forget that the auditory and facial nerves are frequently injured in fracture involving the middle fossa of the base of the skull and implicating the internal auditory meaties; when the nerve is divided permanent deafness will result.

Don't forget that the sixth nerve is more frequently involved in fractures of the base than any other cranial nerve, paralysis of which produces convergent squint.

Don't forget that in fractures of the skull the patient may present no evidence other than those of concussion for days or weeks, and then suddenly develop symptoms of an alarming or fatal character. One such case might be mentioned: A man was rendered unconscious by falling from a car and was taken to a hospital. There were no discoverable evidences of cranial fracture or local brain injury, and the next day as the patient was feeling perfectly well he was discharged from the hospital. He continued well for two weeks, then suddenly died. Autopsy revealed a transverse fracture extending the entire width of the middle fossa.

Don't forget when a person is found unconscious, with paralytic symptoms, with or without scalp wounds, it is not always easy to determine whether the coma is apoplectic in origin, due to an extreme degree of intoxication, or the result of cranial fracture.

Don't forget that while the x-ray is not infallible, it is inaluable diagnostic aid and should be used in all fractures, regardless of the anatomic situation, both before and after reduction.

Don't forget to carefully watch the patient suffering from fracture of the shaft of the femur dressed with circular plaster of Paris, as it may become loos frome recession of the swelling; the patient may so rest in bed that his foot and leg may turn in the plaster and inversion or eversion of the foot occur.

Don't forget to be very careful in the remarks you make to the family or the patient who has a bad result following fracture, lest you be haled into court to testify against a brother doctor or practitioner. I once heard a prominent lawyer say that doctors themselves were responsible for the majority of suits for malpractice.

Don't forget that the majority of suits for malpractice have their origin in imperfect functional results or visible deformities following the adjustment of fractures; therefore in complicated cases the possibility of future impaired function should always be explained to the patient in the presence of competent witnesses.

Don't forget in ambulatory fracture of the lower extremities where crutches are necessary, to give the patient a good fit and pad the crutches well so as to prevent crutch paralysis from pressure upon the musculo-spiral nerve.

FINALLY: Don't forget that eternal vigilance is the price of good results in all fractures.—American Journal of Surgery.

